# Stress at Work: Can the Spiritual Dimension Reduce It? An Approach From the Banking Sector

**DOI:** 10.3389/fpsyg.2021.715884

**Published:** 2021-10-15

**Authors:** Rafael Robina-Ramírez, José Amelio Medina-Merodio, Rosa Estriegana, Marcelo Sánchez-Oro, José Castro-Serrano

**Affiliations:** ^1^Departamento de Dirección de Empresa y Sociología, Universidad de Extremadura, Cáceres, Spain; ^2^Departamento de Ciencias de la Computación, Universidad de Alcalá, Madrid, Spain; ^3^Departamento de Automática, Universidad de Alcalá, Madrid, Spain; ^4^Departamento de Arte y Ciencias del Territorio, Universidad de Extremadura, Cáceres, Spain

**Keywords:** stress, work, transcendence, values, spiritual dimension, banking

## Abstract

Stress at work motivated by pressures and labour control can alter the behaviour of workers. Since the 2008 economic crisis, banking in Spain has suffered a series of massive lay-offs to adjust to the new market situation. This new financial restructuring has meant greater labour pressure to achieve the required results. Faced with this adversity, employees have experienced greater stress at work. This work analyses the effect of reinforcing employees’ spiritual dimension to transcend and correctly manage work pressure and stress at work. In so doing, 601 employees from 294 financial entities of five large IBEX banks participated in this pilot project. Through a participatory methodology based on a review of the literature, the study indicators have been delimited. The data obtained have been treated using the SEM-PLS method. The results propose the incorporation of a series of tools to reinforce values and transcendent employee behaviour.

## Introduction

Due to the 2008 international economic crises in the financial sector, different restructuring processes have been approached, such as that used in the 1929 crises (Reinhart and Rogoff, 2009; Sánchez, 2010; De Haan et al., 2020). In Spain, banking has experienced a 54.4% reduction in financial entities from 2008 to 2020 (CCOO, 2020). High unemployment rates and job insecurity have ended up affecting competitiveness in the banking system (Barroso, 2017; Klutse, 2020). The impact that the closure of offices has had on the well-being, personal stability and health of employees has been devastating (Cortès-Franch and López-Valcárcel, 2014).

According to the new survival scenario within the banking sector, the need to obtain results has become the main pressure on employees (Lin et al., 2012). It erodes the trust between employees and managers (Robina-Ramírez et al., 2020a; Wang et al., 2020; Robina-Ramírez, 2021) and increases stress at work (Altavilla et al., 2020; Sanusi et al., 2020). As a result, it definitely contributes to a gradual worsening of workers’ mental health (Espino Granado, 2014; Barceló et al., 2016; Khalid et al., 2020) and their living conditions (i Meléndez, 2012). According to Suzabar et al. (2020), adverse social and organisational working conditions can turn work into a toxic place not exempt from extreme pressure. This situation leads to states of fear, paranoia, and anxiety (Mausner-Dorsch and Eaton, 2000; De Clercq et al., 2020).

Different authors have approached the phenomenon of stress in the organisational field (Long, 1995; Quick and Cooper, 2003; Gismera et al., 2019; Wang et al., 2020). From the organisational point of view, stress can come from the lack of freedom of decision possessed by employees in jobs with high work demands, facing uncertain employment consequences or long-term job insecurity; over time, these may lead to physical exhaustion (Ganster, 2013).

The origin of stress at work can come from external issues, such as abusive socio-labour conditions (Robina-Ramírez, 2017; Robina-Ramírez et al., 2020d), or from internal ones (Sánchez-Hernández et al., 2020a). It can also result from a lack of personal values that make it difficult to control stressful situations. Rather than analysing the organisational causes of employee stress, we delve into ways to reduce stress at work through developing a transcendent sense in the workplace (Robina-Ramírez, 2021). As a result, a research model is proposed so as to study what role spirituality plays in responding to current working conditions in bank employees. From the well-known model of Schwartz’s behavioural transformation (1973), three factors are highlighted: (1) knowledge of the consequences of an adverse work environment; (2) an attitude built on personal values (Robina-Ramírez and Medina-Merodio, 2019); and (3) the development of transcendent behaviour in employees.

A total of 601 employees from five large banks of the IBEX-35 have participated in the work. Based on a participatory procedure, 24 employees and four managers were involved in the design of the indicators based on two focus groups (Sánchez-Oro Sánchez and Robina-Ramírez, 2020). The study was completed using two training sessions to deepen the importance of values and the transcendent meaning of work; 50% of the total participants were involved in these sessions. Their objective was to convey the role and meaning of spirituality and values in the control of stressful situations.

To date, there is not enough empirical evidence to support a stress control model among employees of the banking sector that goes beyond modifying employees’ working conditions or organisational aspects. This proposal provides training tools to develop spirituality so as to reduce stress at work, hence this approach may arise as a future line of research.

## Theoretical Framework

### Stress at Work

Stress as a psychological reaction of the person to certain stimuli at work has been widely studied (Hendriks et al., 2020). However, this phenomenon is not a mere physiological response to an unexpected situation but rather a reaction in the individual that comes from the interaction between what society demands of them and their response (Long, 1995). The dysfunction between what is expected (assumption of the workload) and what is contributed by the worker (transformation of the workload into results) can lead to a stress reaction. This dysfunction can become a risk factor that could end in physical and mental professional exhaustion through a variety of health disorders and diseases (Quick and Cooper, 2003).

Quick et al. (1997) identified four causes of stress derived from both the work environment and personal characteristics: (1) work overload and job insecurity; (2) conflicts and confusion of the roles to be performed and their relationship with the styles of leadership; (3) physical causes such as temperature, lighting and workplace design; and (4) personal causes due to personality problems or conflicting interpersonal relationships.

In the daily activity of the organisation, five factors can define any stressful situation at work: (1) the role of the employee in the organisation according to their level of responsibility (Murphy, 1995; Carroll and Laasch, 2020; Willness et al., 2020); (2) professional development related to promotions, job security and career development opportunities (Church et al., 2021; Marvel, 2021); (3) interpersonal relationships in the workplace that can lead to conflictive social relationships involving harassment, discrimination and threats (Greer, 2021; Pellegrini et al., 2021; Vila-Vázquez et al., 2021); (4) the organisational climate in relation to management style, the execution of work tasks and their performance and the type of participation in planning (Robina Ramírez, 2017; Jacobs and Kaufmann, 2021; Knight et al., 2021; Su et al., 2021); and (5) lack of rewards for work done (Siegrist, 1996; Balducai et al., 2021).

These factors are activated in circumstances of crisis, namely the crisis that the banking sector has faced since 2008 with devastating consequences for employees. In addition, massive lay-offs (Tarki et al., 2020), high results control systems in financial operations (Altavilla et al., 2020), increasing competitiveness to attract financial resources (Klutse, 2020), long working hours until objectives are reached (Sanusi et al., 2020) and high turnover in positions and offices (Sudria, 2016; Kurdi and Alshurideh, 2020; Yukongdi and Shrestha, 2020) have been identified.

### The Spiritual and Transcendent Dimension in the Face of Adverse Conditions at Work

Adverse working conditions currently faced by banks in Spain, characterised by the fragility of the Spanish banking system (García and Vázquez, 2019) and high temporality (Araújo-Vila et al., 2020), is translated into aggressive client deposit-taking policies (Royo, 2020). This hinders employees’ personal and vital stability.

Nowadays, the psychological conception of suffering is managed by providing tools to regulate emotions at work (Robina-Ramírez and Pulido-Fernández, 2019; Robina-Ramírez et al., 2020b). In the business scenario, the role of transcendence and spirituality is playing a key role to appropriately channel adversity (Gupta et al., 2014; Counted et al., 2020; Sanchez-Hernández et al., 2020b; Vasconcelos, 2021).

The topic of spirituality is interdisciplinary, intercultural and rich in giving meaning to suffering (Weathers et al., 2016). While some authors relate it to the search for the “sacred” (Zinnbauer and Camerota, 2004), others identify it with human qualities that facilitate a transcendent vision to bring new meaning to human actions beyond the materiality of work (Reischer et al., 2021).

According to the European Forum, Spirituality in Economics and Society (European Forum SPES, 2014), transcendence is aligned with the search for the deep meaning of human actions connected with the sacred, with the excellent and with the perfection to which every person can aspire (European Forum SPES, 2014). The sacred contains an idea of divinity, beauty, perfection and goodness, which has its origin in Creation (Ellingson, 2016). This inspiring reality transcends human realities, and an idea of creation lies at the base of any work, namely a more habitable, better organised and shared world (Robina-Ramírez and Cotano-Olivera, 2020; Robina-Ramírez et al., 2020c).

Translating the idea of perfection to organisations, a principle of transcendence in the way of doing any works can be discovered. This principle can be introduced in the designing, organising and evaluating process according to the premises of perfection and goodness (Lepisto and Pratt, 2017). Transcendence allows employees to provide directionality at work under the premises of beauty, perfection and kindness, which allows them to overcome unexpected situations at work and reduce their harmful effects (De Clercq et al., 2020).

In managing the unexpected situations which can increase the gap between what society demands of the individual and their response, emotions, attitudes, and conscience come into play. Spirituality helps to give direction and prioritise each of them based on a pattern developed by the individual themselves from their beliefs and values (Vasconcelos, 2021).

Setting priorities involves developing an internal analysis, setting goals and above all looking towards what is more perfect than us; this allows us to receive inspiration to grow and improve. Inspiration comes when personal models are imitated through aspirations and a desire for growth. As a result, following these personal models is possible to overcome egocentric, static and self-centred behaviours so as to initiate the search for meaningful work to identify what role an individual plays in both professional environments and society.

The development of this role provides tools to the employees to overcome pressurising situations and reduce their work stress. Work stress is lessened when employees’ objectives go from mere economic and financial gain to the incorporation of adversity as part of the integral development of the person. In other words, when meaningful work is added to the daily management through adequate responsibility especially, when adversities are addressed as growth opportunities for the benefit of the organisation and third parties.

At this point, our hypotheses are proposed.

*H1*: The development of transcendent behaviour (CT) influences a reduction in work stress (RWS).

*H2*: Knowledge of the consequences of an adverse work environment (CA) influences a reduction in work stress (RWS).

*H3*: Knowledge of the consequences of a CA influences the development of transcendent behaviour (CT).

### The Development of Values in the Face of an Adverse Work Environment

According to Norris (2019), any change in people’s attitudes occur thanks to the monitoring of behavioural patterns. Exemplary personal performance develops a mimetic effect that inspires individuals to transform their behaviours. However, any change in behaviour needs the support of values to bring this change into effect (Finegan, 1994). This mimetic effect is also transferred to the organisation in each of its activities.

In adversity at work, the acquisition of values endows the person with a sense of calm and tranquillity that allows them to control emotions through the exercise of patience (Schnitker, 2012), compassion (Lim and DeSteno, 2016) and forgiveness to face injustices (Flaskas et al., 2007). These values allow individuals to develop positive feelings to be tolerant, respectful at work (Mazzola and Kessler, 2012) and supportive (Wilmers, 2019).

The embodiment of those values not only gives rise to authenticity and integrity at work (Meyer and Parfyonova, 2010) but also guides behaviour towards successful experiences at work (Schwartz, 2007; Ménard and Brunet, 2011). Furthermore, it helps to cultivate hope for what is to come (Anandarajah and Hight, 2001), as well as gratitude to any opportunity to combat any adversity (Wood et al., 2010).

In this process of behavioural transformation, we find different leaders who have embodied those values in their lives. As a result, they are an example towards facing adversity (Cartledge, 1977; Kearns, 1989; Keen, 2020; Le Goff, 2020; Stokes-Parish et al., 2020). The characters or heroes are characterised by having sought the interest of others before their own, and therefore, they become a CT model to follow (Winter, 1994). Values that decisively contribute to social and personal change, as well as to the motivational bases of attitudes and behaviour, emerge in the narratives of their lives (Schwartz, 2012).

We can thus state the following hypotheses:

*H4*: Knowledge of the consequences of a CA influences the acquisition of values (V).

*H5*: The acquisition of V influences the development of CT.

*H6*: The acquisition of V influences the RWS.

### Working Model and Hypothesis

*H1*: The development of CT influences the RWS.

*H2*: Knowledge of the consequences of a CA influences the RWS.

*H3*: Knowledge of the consequences of a CA influences the development of CT.

*H4*: Knowledge of the consequences of a CA influences the acquisition of V.

*H5*: The acquisition of V influences the development of CT.

*H6*: The acquisition of V influences the RWS.

**Figure 1:**
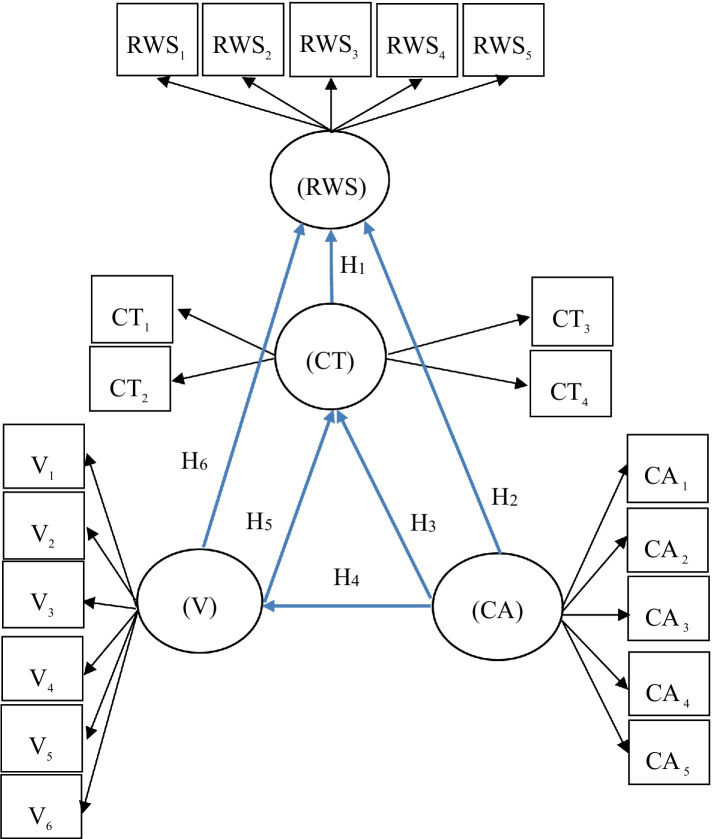
Presentation of the model.

Figure 1 shows the model and the hypothesis.

## Methodology

### The Behaviour Transformation Model Based on the Schwartz Model

It was Schwartz who in 1973 proposed a widely tested model to study the behaviour transformation process (Schwartz, 1973). This model explains the change in final behaviour. This is made from the knowledge of the consequences of an action (awareness of the consequences: CA). This generates responsible behaviours [assignment of responsibility (AR)] and personal obligations of action [personal norms (NP)].

As Schwartz and Howard (1981) pointed out, the behaviour transformation process develops moral actions to execute or avoid responsible actions. This process follows the next steps: the knowledge of the consequences of a CA (awareness of the consequences) can lead to responsible actions through the development of V (AR) to generate norms that transform behaviours (CT) (through NP) that influence behaviours that reduce the work stress generated by work (RWS).

### Population and Sample

Based on employees employed in the five large banks of the IBEX 35 (see Table 1), 273 financial entities belonging to these banks were randomly chosen, distributed among the 15 cities with the largest population in Spain.

**Table 1:** Mergers in the banking system in Spain as of 2008.

BANCO SANTANDER	Banco Central Hispano	Banco Central, Banco Hispano Americano
	Banco Santander	Banesto, Banif
	Banco Popular	Banco Pastor, Banco de Andalucía
BBVA	Unnim	Caixa Sabadell, Caixa Manlleu, Caixa Terrasa
	BBVA	Catalunya Banc
	Catalunya Banc	Caixa Tarragona, Caixa Manresa, Caixa Catalunya
CAIXABANK	Banca Cívica	General de Canarias, Cajasol, Caja de Burgos
		Caja Navarra; C. Guadalajara
	CaixaBank	Barclays, Caixa Gerona, Banco de Valencia, Banco de Valencia
BANKIA	BFA Bankia	Caja Madrid, Bancaja, Caja Rioja, Insular de Canarias
		Caja Ávila, Caja Segovia, C. Laietana
	BMN (Banco Mare Nostrum)	Sa Nostra; Caixa Penedés, Caja Murcia, Caja Granada
BANCO SABADELL	Banco Sabadell	CAM, Banco Guipuzcuano
	Banco Gallego	

Source: own elaboration based on CCOO (2020). Report on the evolution of employment in the financial sector restructuring process 2008–2020, CCOO Servicios, Madrid.

During the 1st week of February 2021, the research team contacted the director of each entity by telephone after sending a cover letter. Throughout the month of February, adhesion emails were received either from the office director or from employees interested in participating individually. In the 1st week of March, participation reached 632 employees, of which 31 left the study for personal reasons. The final sample was 601 employees from the Spanish banking sector (Table 1).

The majority of the participants were male (55%), aged between 30 and 39 years and possessed a university education (57%; Table 2).

**Table 2:** Sample composition.

	*N*=601	%
Gender		
Male	330	0.55
Female	269	0.45
No responded	2	0
Total	601	1
Age		
18–29	136	0.22
30–39	164	0.27
40–49	153	0.25
50–59	103	0.2
More than 60	39	0.06
No responded	6	0.01
Total	601	1
Level of studies		
Primary education	6	0.01
Secondary education	60	0.1
Bachelor	95	0.16
University	343	0.57
Master	91	0.15
No responded	6	0.01
Total	601	1

### Selection of Indicators

In the book “grupos focales como herramienta de investigación turística” “focus group as a tool for touristic research,” Sánchez-Oro Sánchez and Robina-Ramírez (2020) explains that over the past decade, focus groups and group interviews have re-emerged as a popular technique for gathering qualitative data, both among sociologists and across a wide range of academic and applied research areas, such as tourism.

Focus groups are currently used as both a self-contained method and in combination with surveys and other research methods, most notably individual, in-depth interviews. Comparisons between focus groups and both surveys and individual interviews help to show the specific advantages and disadvantages of group interviews, concentrating on the role of the group in producing interaction and the role of the moderator in guiding this interaction. The advantages of focus groups can be maximised through careful attention to research design issues at both the project and the group level. Important future directions include: the development of standards for reporting focus group research, more methodological research on focus groups, more attention to data analysis issues, and more engagement with the concerns of the research participants.

Due to the role that spirituality plays on providing solution to reduce the stress at work, we have chosen this technic so as to discuss the indicators just at the beginning of the research process. Contrasting the variables and indicators with employees and managers have helped us to understand their points of view about the questions we have made to them.

At the beginning of the research design 250 employees and 50 managers were randomly invited to be part of the focus groups (see Table 3). Emails were sent to them explaining the scientific proposal of the research as well as the methodology we followed which is based on two steps. Eventually 24 employees and 6 managers were part of the work team. In the first one we carefully explained the main concepts of the research work: Schwartz activation norm model, stress at work, values, spirituality, transformation of the conducts. For about 2h, we received suggestions about how these topics had to be translated to the banking sector. In the Table 1, it is shown the distributions of participants from each regional bank.

**Table 3:** Distributions of participants in the collecting data procedure.

	Initially proposed randomly		Proposal accepted	
	Employees	Directors	Employees	Directors
SANTANDER BANK	50	10	4	1
BBVA	50	10	6	2
CAIXABANK	50	10	3	1
BANKIA	50	10	7	1
SABADELL BANK	50	10	4	1
	250	50	24	6

During the 2nd week of March 2021, two focus groups were held through the online platform Zoom to select the indicators for each of the latent variables extracted from the literature review. A total of 24 employees and six managers were part of the work team. In the first meeting, the concept of each of the indicators extracted from the literature review was transferred. The typology of adverse situations and the values necessary to develop CT were analysed so as to reduce the stressful work in the banking sector. During the second meeting, the meaning of the indicators was examined and modified. The results are shown in Table 4.

**Table 4:** Indicators.

Indicators		Authors
RWS: Reduction in work stress		
(RWS1)	Reduce the stress produced by the level of responsibility and different roles	Murphy, 1995; Carroll and Laasch, 2020; Willness et al., 2020
(RWS2)	Reduce stress caused by a lack of promotion, job security and career development opportunities	Church et al., 2021; Marvel, 2021
(RWS3)	Reduce stress caused by negative interpersonal relationships within the workplace	Greer, 2021; Pellegrini et al., 2021; Vila-Vázquez et al., 2021
(RWS4)	Reduce stress produced by a toxic organisational climate	Jacobs and Kaufmann, 2021; Su et al., 2021
(RWS5)	Reduce stress produced by the absence of rewards for the work done	Siegrist, 1996; Balducai et al., 2021
CT: The development of transcendent behaviour		
(CT1)	The behaviour is linked to a transcendent attitude in their work that goes beyond mere financial gain	Davis, 2015
(CT2)	Spirituality modifies the perception of clients’ desires and aspirations	Kochukalam and Srampickal, 2018
(CT3)	Transcendence and spirituality can become the main engine of the daily routine	Lerner et al., 2006
V: Values		
(V1)	Compassion towards employees and clients in adverse circumstances	Lim and DeSteno, 2016
(V2)	Developing an attitude of reconciliation between colleagues would increase their happiness	Flaskas et al., 2007
(V3)	Developing hope for what is to come would increase their happiness	Schnitker, 2012
(V4)	Tolerance and respect at work	Mazzola and Kessler, 2012
(V5)	Solidarity	Wilmers, 2019
(V6)	Authenticity and integrity	Meyer and Parfyonova, 2010
CA: Knowledge of the consequences of an adverse work environment		
(CA1)	Mass lay-offs	Tarki et al., 2020
(CA2)	High fragility of the Spanish banking system	García and Vázquez, 2019
(CA3)	High temporality	Araújo-Vila et al., 2020
(CA4)	Aggressive customer acquisition policies and demanding work schedules	Royo, 2020
(CA5)	High mobility between offices	Sudria, 2016

### Training Sessions

Once the indicators were defined, the research team held two voluntary training sessions for the participants. The first was entitled “The role played by transcendence in behaviour modification” and the second “Values as axes on which to build a change in behaviour.” Approximately 50% of the total participants agreed to join the training sessions given in the 4th week of March using Zoom. In both sessions, examples were selected from a group of characters who have stood out for their personal qualities, as well as narrative passages that have correctly described the overcoming of adversity thanks to their values and their capacity for transcendence.

Table 5 lists the characters or written passages highlighting exemplary behaviours and personal values. These were grouped into four sections (relational, authenticity, justice, and forgiveness). From the carefully selected passages, a connection was established in the training sessions between the adversities in the current jobs of bank employees and a new way of understanding their work based on the improvement of spirituality and meaning of work.

**Table 5:** Authors and passages that have correctly described the variables of the model.

Variables	Characters-narratives	Exemplary behaviours
Behavioural changes		
Search for the meaning of life	Viktor Frankl (1905–1997) (Längle and Sykes, 2006) (Yunus et al., 2010).	Human capacity to transcend the difficulties of a concentration camp The value of sincerity that helps to develop in a person and those around them
Origin of creation	Bible (Genesis 1–2) (Wenham, 1994).	Communicate inner goodness to other creatures Discover the reality of providence in people and government of creatures
Contemplation of the beauty of creation	Bible (Genesis 1–2) (Wenham, 1994).	Learn from the fruits of the contemplation of beauty Develop acts of gratitude and praise
Values		
Relational (kindness, respect, tolerance)	Mahatma Gandhi (1869–1948) (Barnabas and Clifford, 2012).	Sowing peace, friendship and tolerance as a way of life Respect for the ideas of others
Authenticity (honesty, trust, fidelity, sincerity and optimism)	Marco Tulio Cicerón (106 aC-46 aC) (Sierra, 2009).	Honesty must be the ultimate goal to which a human being must aspire above the useful and pleasant Honesty is one of the most important components of a healthy personality.
Justice (solidarity, freedom and commitment)	Muhammad Yunus (1940–2018) (Yunus et al., 2010).	Development of a micro-credit programme in poor neighbourhoods Committed to the extension of solidarity entrepreneurship
Forgiveness, reconciliation with an adversary	Teresa de Calcutta (1910–1997) (Poplin, 2011).	They met all those who have experienced pain and suffering They practised mercy in all their actions
Forgiveness, patience in the face of adversity	Nelson Mandela (1918–2013) (Glad and Blanton, 1997) Martin King (1929–1968) (Ward et al., 1996).	Faithful fighters to achieve peace in South Africa before the yoke of whites and an example of learning how to keep a country at peace in the midst of differences Luther King patiently fought to realise the American dream to advance the rights of African Americans
Tenacity and perseverance in the face of adversity	Marie Curie (1867–1934) (Radvanyi and Villain, 2017).	Curie managed to stand out with tenacity and effort in a time where machismo and xenophobia were present everywhere. She was the first person to receive two Nobel prizes: one for Physics in 1903 and one for Chemistry in 1911.

Once the training sessions were finished, 18 participants in said sessions were selected to analyse the validity of each of the questions in the questionnaire. Three of the questions were modified.

### Tabulation of Data From PLS-SEM

PLS-SEM is an exploratory methodology that is based on primary or secondary data, which makes it ideal for approaches in which the objective is oriented to researchers’ predictions, since it does not require a normal distribution of data and adapts to small sample sizes (Chin and Newsted, 1999). PLS-SEM also provides R2 values and indicates the importance of relationships between constructs and can handle numerous independent variables at the same time (Hair et al., 2011). Bootstrapping-based methods are used to evaluate the general fit of the model in PLS, which seems to work quite well, as indicated by Dijkstra and Henseler (2015).

This statistical technique is observed when dependency relationships are established between latent variables and indicators (Sarstedt et al., 2016). For the generation of the statistical model, the PLS (Partial Least Squares) SmartPLS 3 Version 26 technique was applied. This version is particularly recommended for composite site models (Rigdon et al., 2017). SEM-PLS modelling was defined based on two approaches: the measurement model and the structural model. To proceed with the analysis of the structural model, the reliability that exists between the indicators and the constructs was analysed, as well as the validity of the measurement model (Hair et al., 2011). In this case, we use reflective elements because they are interchangeable (Haenlein and Kaplan, 2004).

### Evaluation of the Fit of the Global Model

Although the criteria proposed to fit the model in PLS-SEM are in the initial stage of investigation, they should be taken with some caution (Hair et al., 2017). However, when applying a consistent PLS approach (PLSc-SEM), focused on reflexively measured constructs, one may be interested in the fit of the model. Therefore, it is possible to mimic CB-SEM more completely through the PLSc-SEM approach (Henseler et al., 2016; Sarstedt et al., 2016). SmartPLS offers the following fit measures: firstly, the residual standardised root mean square (SRMR), which is defined as the difference between the observed correlation and the implicit correlation matrix of the model, so values less than 0.08 (Hu and Bentler, 1998) and Henseler et al. (2016) suggest that SRMR <95% starting quantile (SRMR HI95) is considered a good fit (Table 6).

**Table 6:** Measure of fit according to the SRMR.

SRMR	Original sample (O)	Sample mean (M)	**95%**	**99%**
Saturated model	0.013	0.011	0.015	0.017
Estimated model	0.013	0.011	0.015	0.016

Second, there is the normalised fit index (NFI), whose measure represents an acceptable fit above 0.9 (Henseler et al., 2016). In our case, NFI=0.927. The third exact fit of the model is bootstrap-based statistical inference tests, which can be evaluated using the squared Euclidean distance (dULS) and the geodetic distance (dG) as the two different ways to calculate the discrepancy between the empirical covariance and the covariance implicit in the composite factor model (Dijkstra and Henseler, 2015).

Furthermore, Henseler et al. (2016) suggest that dULS and dG should be lower than the starting quantile of 95% dULS<starting quantile of 95% (HI95 of dULS) and dG lower than 95% starting quantile (HI95 of dG; Table 7). Therefore, a model fits well if the difference between the implicit correlation matrix in the model being tested and the empirical correlation matrix is so small that it can be attributed purely to sampling error, hence the difference between the correlation matrix implicit in our model and the empirical matrix. The correlation matrix must be non-significant (*p*>0.05).

**Table 7:** Measure of fit according to the dULS and the dG.

dULS	Original sample (O)	Sample mean (M)	**95%**	**99%**
Saturated model	0.007	0.006	0.010	0.013
Estimated model	0.007	0.005	0.009	0.012
**dG**	**Original sample (O)**	**Sample mean (M)**	**95%**	**99%**
Saturated model	0.013	0.012	0.020	0.025
Estimated model	0.013	0.012	0.021	0.026

## Results

### Data Analysis and Measurement Model

The data have been processed using the multivariate PLS technique. This approach is recommended for social science analysis (Fornell and Bookstein, 1982) in small samples (Hulland, 1999). According to Carmines and Zeller (1979), individual reliability must be analysed right at the beginning of the methodological process of determining the appropriate indicators. In this sense, the loads (*λ*) must be greater than 0.707 (Table 8).

**Table 8:** Outer model loadings.

	CA	RWS	CT	V
CA1	0.928			
CA4	0.900			
CA5	0.929			
RWS1		0.939		
RWS3		0.944		
CT1			0.928	
CT2			0.935	
V2				0.951
V3				0.941

Table 9 examines the individual reliability of Cronbach’s alpha (Nunnally and Bernstein, 1994) and Rho_A with values >0.70 (Dijkstra and Henseler, 2015) and the composite reliability with values >0.5 (Fornell and Larcker, 1981). The minimum level of mean variance extracted (AVE) explains that all the analysed constructs were reliable with more than 50% of variance between their own indicators (Hair et al., 2017). In our case, all the constructs exceed the minimum values of compound reliability and convergent validity.

**Table 9:** Reliability and validity.

	Cronbach’s alpha	rho_A	Composite reliability	AVE	Fornell-Larcker criterion			
CA	0.908	0.910	**0.942**	**0.844**	0.919			
RWS	0.872	0.873	**0.940**	**0.886**	0.723	0.941		
CT	0.847	0.848	**0.929**	**0.867**	0.687	0.680	0.931	
V	0.883	0.888	**0.945**	**0.895**	0.669	0.677	0.682	0.946

To analyse the discriminant validity, the analysis demonstrated by Fornell and Larcker (1981) is carried out by examining the amount of variance that exists between its indicators (AVE), which must be greater than the variance that said variable shares with others in the model (Roldán and Cepeda, 2016). A second, more rigorous analysis is called heterotrait-monotrait (HTMT) whose value must be <0.90 (Henseler et al., 2015; see Table 10). In our case, the criterion is met.

**Table 10:** HTMT ratio.

	CA	RWS	CT	V
CA				
RWS	**0.811**			
CT	**0.782**	**0.791**		
V	**0.745**	**0.771**	**0.786**	

To assess the best fit of the model, it must be analysed using the value obtained from the residual mean square root (SRMR). In our case, the value was 0.042, which did not exceed the approved 0.08 (Hu and Bentler, 1998).

### Structural or Internal Model Analysis

After ensuring that the relationships between the constructs and indicators were acceptable, the structural or internal model was evaluated by examining the relationships between constructs to predict the viability of the model (Hair et al., 2014). The most important value is the coefficient of determination that measures the explanatory capacity of the dependent variable (R2; Hair et al., 2014). This can be weak, moderate or strong, depending on the values obtained (0.19, 0.33, and 0.67, respectively; Chin, 1998). As can be seen in Table 9, the value of R2 is 0.553, which shows that the model is significant and adequately explains the elements that contribute to improving the happiness of bank employees by developing behaviours that reinforce their spirituality.

To measure the path coefficients, we use two nonparametric tests in Table 11: *t* values and value of *p*. Both indicate whether the hypotheses were supported. In this case, the Student *t* value should exceed 1.96 (Hair et al., 2014) and the value of p should be less than 0.05 (Henseler et al., 2009). Both criteria are met in all hypotheses. The path coefficients (*β*) and the t distribution explain the importance of the hypotheses when activating 5,000 subsamples in the original model. This method is called bootstrapping, and it provides the fit of the model (Henseler, 2017). The confidence intervals and *t* values outline a second test to assess the significance of the path coefficient. The measure is based on analysing each interval, which cannot contain a 0 value (Henseler et al., 2009; see Table 11).

**Table 11:** Results of the structural model.

	Path coefficients (*β*)	Low interval	High interval	*T* Statistics	*p*
H1: CT→RWS	0.273	0.141	0.408	3.388	0.000^***^
H2: CA→RWS	0.414	0.260	0.563	4.512	0.000^***^
H3: CA→CT	0.440	0.324	0.559	6.174	0.000^***^
H4: CA→V	0.744	0.677	0.803	19.361	0.000^***^
H5: V→CT	0.459	0.331	0.577	6.242	0.000^***^
H6: V→RWS	0.247	0.095	0.390	2.688	0.004^**^

* *p*<0.05 (t (0.05; 499)=1.64791345).

** *p*<0.01 (t (0.01; 499)=2.333843952).

*** *p*<0.001 (t (0.001; 499)=3.106644601).

### Use of Predictive Analysis

Table 12 also measures Q2 predictive relevance by predicting endogenous latent constructs. The resulting parameter must be higher than Q2>0. In our case, Q2>0.438; this criterion is thus validated.

**Table 12:** Coefficients and parameters of the route.

	*R* ^2^	*Q* ^2^
CA		
RWS	0.616	0.537
CT	0.562	0.484
V	0.447	0.394

## Discussion

In the last decades, banks have faced enormous changes in the structure and organisation. New technology and deregulation labour markets have reshaped working lives by periodically changing working conditions (Kaur et al., 2017). As a result, not only companieś structure and organisations have evolved but also have impacted on working population’s health and their psycho-social disorders of employees. Those changes have had serious effect on bank employees as well as in their daily lives based on the banking sector’s ongoing competition (Bozdo and Kripa, 2015).

The banking sector evolution has defined their task as bank sellers rather bank employees subjected under a great pressure to meet the bank’s objectives by selling investment funds, bonds and insurance policies (Adrian and Ashcraft, 2016). Pressure, tension, and stress in the bank atmosphere is a daily routine that is currently threatening the employee’s banking activity (Silva and Navarro, 2012).

That physical and mental experiences related to the stress at work were analysed through the interviews made to employees and directors in the banking sector. Among them “Workers are under immense levels of pressure, from both the demands of the job and the knowledge that automation might soon put them out of work. As a result, far too many days are lost to stress every year” (employee 2), “when you consider the effect that stress will have on employee productivity, the figure’s likely much, much higher” (employee 4), “if you talk to every employee, you might start to hear the same issues come up again and again.” At this point, you’ll have a good idea of the biggest causes of stress in your organisation. And once you know what’s making your employees stressed, you can start thinking about the steps you could take to make things better (employee 7), “for us stress can cause headaches, heartburn, insomnia, depression, and other serious conditions. In the short-term, this will make your team unhealthy, unhappy, and unproductive. It’s no wonder that so many days are lost to stress each year” (employee 11), “stress is a normal human reaction. The financial sector is defined by risk and high-stakes decisions. Of course, workers are going to feel stressed. But when the stress gets too much, when it starts to affect workers’ health and, by extension, the business, then you have a problem” (employee 14), “when it comes to organisational change and restructuring, it’s not necessarily the change itself that causes stress. It’s the uncertainty. Employees hate the idea that 1day they could show up to work to find that everything’s changed or worse, that they do not have a job anymore. This must cause millions of sleepless nights each year” (employee 18).

Similarly, director also have shown what the stress is harming to employees and the managing system at the banking sector. “Leaders and managers are important role models in fostering healthy behaviours at work. These findings underline how harmful the impact can be if managers aren’t equipped with the competence and confidence to go about their people management role in the right way” (director 1), “if you foster a community that’s entirely driven by profit and results, do not be surprised when the air turns toxic. This sort of attitude could turn your employees against each other” (director 2), “businesses work best when everyone works together towards a shared goal. If this goal is nothing more than ‘profit,’ then you may create an unhealthy culture of ruthless competition. This is bad for stress levels and very bad for productivity” (director 4).

The “Report on the evolution of employment in the financial sector restructuring process 2008–2020” prepared by CCOO (2020) for the banking sector in Spain. It proposes mechanisms to improve working conditions based on external issues, such as the development of a greener, more sustainable and digital economy, improving ethical banking and guaranteeing financial inclusion in an environment with fewer operators, fewer offices and fewer staff.

However, they put aside the personalised training in values and the transcendent vision of work, which gives them a more complete meaning of adverse conditions based on exemplary behaviours of individuals who have excelled throughout humanity.

In the last decades, spirituality as a searching process for transcendence, entails abilities drawn from spiritual themes to improve and better adapt workers to pressure and stressful tasks at the company (Emmons, 1999). Those adaptation comes from the developing of values and personal capabilities which can place the actions and lives in a wider, richer, meaningful context (Zohar et al., 2000).

Spirituality provides a deep understanding about question that goes beyond professional issues increasing the awareness of developing an adaptive application of the non-material and transcendence aspects of one’s existence (Vaughan, 2002). Spirituality not only provides a transcendental awareness about a meaningful perception of the self, of other and the physical world also implies a personal meaning production by developing the ability to construct purpose in physical and mental experiences (King and DeCicco, 2009).

Leadership spirituality intrinsically motives and drives the employees’ internal locus towards organisational development by aligning their personal values with the values of organisational vision and mission which resultantly creates a psychological affiliation between employees’ and their organisations and increases their level of self-fulfilment at work.

Personal values are highlighted in the life of outstanding individuals. Based on those values two training sessions were given in order to make them see the possibility of transforming behaviour based on the ability that each employee and manager has to transcend adverse conditions (Viktor Frankl, 1905–1997) and develop acts of gratitude to the good received (Bible, Genesis 1–2).

According to Markow and Klenke (2005) leadership spirituality not only impart delight full impact on the job performance, productivity and organisational attachment, but also affiliation and commitment with a decline in employee’s turn over tension and stress. In the same direction, Fry et al. (2003) highlight that values emphasise on innovation, affection, interconnectedness and better communication which reduce the stress at work.

For this, an empirical model has been presented in order to reinforce the spirituality and transcendence of employees in their daily activities. This model is based on the knowledge of adverse conditions and of the values that are necessary to incorporate to adapt the response of employees to adverse conditions in order to be able to reduce work stress.

From the results obtained, we observe that all the external loads of the indicators elaborated by the consensus of the bank employees are valid (*λ*>0.7). To achieve this result, a participatory methodology was developed not only in the definition of the indicators but also in the organisation of training actions to deepen the factors and indicators that contribute to reducing work stress.

The model presented is significant with a moderate-high explanatory capacity (*R*^2^=0.616). Following Schwartz (1973), it can be said that knowledge of CA leads the employee to need to develop a series of values as the main means to combat them (V) (H3: CA → V; *β*=0.744; *T*=19.361). The values have been extracted from the examples of: Mahatma Gandhi (1869–1948) “tolerance and respect for others”; Marco Tulio Cicero (106BC-46BC) “Honesty”; Muhammad Yunus (1940–2018) “commitment and solidarity”; Teresa of Calcutta (1910–1997) “compassion”; Nelson Mandela (1918–2013), Martin Luther King (1929–1968) “reconciliation”; and Marie Curie (1867–1934) “patience.” The teaching of these values has helped to develop CT among bank employees (RE) (H5: V → CT; *β*=0.459; *T*=6.242). The result opens the doors to a RWS among employees (H1: CT→RRWS; *β*=0.273; *T*=3.388). All those examples have personified values which have become a meaningful insight for the employees. It has also been stressed by Giacalone and Jurkiewicz (2003) adding that beside focusing on the values of organisational vision of the company the employees’ self-fulfilment need should also be given coherent importance to personifies strong commitment and organisational accomplishment as a way to reduce tensions at work.

Those theories are well explained through Ryan and La Guardia (2000) who say that the organisations with cultures reflecting spiritual values such as individual support, effective communication, coordination, collaboration and strong interpersonal relationships which motivate employees and reduce tensions at work. Other benefits of developing those values are pointed out. According to his view employees’ effectiveness reduce absenteeism by creating a cognitive attachment to remain loyal and careful for the future development of the organisation. That cognitive and psychological association with their organisations strengthens their commitment at work with strong sense of fulfilment which, eventually channel any potential dimension of stress in the company (Lilius et al., 2005).

Regarding to those statement drawn from the literature review, managers (D) and employees (E) were asked about the chosen mode to reduce work stress. Two modes were proposed: (1) direct; from the knowledge of the adverse consequences in advance or (2) indirect; following the steps proposed by the Schwartz model from the promotion of V and CT.

Six dichotomous questions were added at the end of the original questionnaire with answers (Yes/No/Do not Know). Of the total sample (*n*=601), 496 are employees (E) whose response percentage was 72% and 106 managers (D) who answered 77% of the questions.

As can be seen in Table 13, both employees and managers prefer to reinforce values and their CT as mechanisms to reduce work stress, since the former presents higher values than the latter. Results are aligned with Frew (2000) when he states a view that spirituality is found beneficial in reducing the feelings of tensions, anxiety as it aids the employees to cope with the burden and pressure of work.

**Table 13:** Direct and indirect mechanisms to reduce work stress (*n*=601).

Schwartz model: CA-V-CT-RWS	E	D
Do you think that the development of V and CT could help you reduce the work stress caused by a layoff?	9.5	9.1
Do you think that the development of V and CT could help you reduce the work stress produced by aggressive customer acquisition policies?	9.1	8.9
Do you think that the development of V and CT could help you reduce the work stress produced by a mobility plan between offices?	8.3	8.5
Direct effect (CA-RWS)	E	D
Do you think that the knowledge of a possible dismissal well in advance could help you to directly reduce the stressful situation that it may cause you?	7.9	7.5
Do you think that knowing about aggressive customer acquisition policies well in advance could help you to directly reduce the stressful situation that it may cause you?	7.5	7.3
Do you think that knowing the mobility plan between offices well in advance could help you to directly reduce the stressful situation that it may cause you?	6.4	6.1

## Conclusion

Three conclusions can be drawn from the study results:

First: Not only were all the hypotheses validated (H1–H5; value of *p*<0.05), but the proposed model also allows us to establish a training strategy to go beyond the routine work of each bank employee and thus to propose modes of overcoming suffering at work. Based on the model proposed and the hypotheses validated the training strategy is based on turning the workplace in a place where elements of spirituality have an impact on an organisation since the organisation is able to gain advantages by developing a humanistic environment in which workers can achieve their full capacity.

As a result of the focus groups statements as well as studies drawn from the literature review stressed in the paper we believe that a humanistic work environment can create a win-win situation for employees, for employee’s coworkers, reducing stress and tension in the workplace and that it is good for the banking sector. If the employees are at liberty to bring his or her physical, intellectual, emotional, and spiritual attributes to the workplace, they will become more productive, creative, and fulfilled. On the contrary, if the employees work in a dispirited workplace, they will manifest themselves in various work troubles- low morale, rising absenteeism, high turnover, burnout, frequent stress-related illness and non-committed to the organisation. Hence, the purpose of the training strategy is to give knowledge about the association between workplace spirituality (team’s sense of community, alignment between organisational and individual values, sense of contribution to society and enjoyment at work) and affective commitment among employees and directors who are working in the banking sector in Spain.

Second: Employees and managers in the banking sector in Spain opt for a model to reduce work stress based on the learning of behavioural models based on the development of values and a transcendent vision. So, unlike the social improvements proposed by CCOO (2020) in the “Report on the evolution of employment in the financial sector restructuring process 2008–2020” based on organisational issues, this model proposes improvements that lie in the development of values and the transcendent vision of work to overcome adverse conditions resulting from massive lay-offs (Tarki et al., 2020), high fragility in the Spanish banking system (García and Vázquez, 2019) and high temporality (Araújo-Vila et al., 2020), aggressive policies to attract clients, demanding work schedules (Royo, 2020) and high mobility between offices (Sudria, 2016) which hinders personal and vital stability.

What the model has proved is that employees and director convey the intention to reduce the stress at work by developing their spirituality in the workplace based on values and transcendence. According to Morales-Sánchez and Cabello-Medina (2015) we are working on developing the values that will be incorporated in the second study that is about to start. It is based on presenting a list of values from that previous research such as: amiability, commitment, courage, environmental responsibility, generosity, gratitude, honesty, humility, justice, optimism, perseverance, prudence, self-control, service to others, solidarity and transcendence.

Third: The proposed model allows the construction of a set of training actions based on the contributions already made by international leaders who have stood out for their values, spiritual resources and ability to transcend at work. They include political leaders, scientists, religious leaders, economists, historians and humanists.

Fourth: According to the *R*^2^ is 0.553, the model shows that is significant and adequately explains the elements that contribute to improving the happiness of bank employees by developing behaviours that reinforce their spirituality. That result is based on the happiness management which explains how workers from the banking sector are searching for welfare through quality, health, and safety parameters (Ravina-Ripoll et al., 2021).

The main limitation of this work has been the difficulty in organising joint variable selection sessions such as training sessions. For this reason, we have had to multiply the number of sessions to reach the largest number of bank employees. This has caused a loss of information when verified by several reporting agents at the same time.

Among future lines of research, the research team is working on data capture to develop a longitudinal model in order to validate the effect of training in values and significance in improving the capacities of bank employees to respond to stressful situations. An action plan is proposed to reduce the level of employeeś stress in the banking sector by developing personal and professional values. That Project will be incorporated in the second study. It is based on presenting a list of values such as: amiability, commitment, courage, environmental responsibility, generosity, gratitude, honesty, humility, justice, optimism, perseverance, prudence, self-control, service to others, solidarity and transcendence. Those values will be tested in the banking sector in order to set up indicators to be applied at the workplace.

## Data Availability Statement

The original contributions presented in the study are included in the article/supplementary material; further inquiries can be directed to the corresponding author.

## Author Contributions

RR-R, JAM-M, and RE wrote the paper and retrieved the data. MS-O and JC-S supervised the paper and included new literature. All authors contributed to the article and approved the submitted version.

## Funding

The publication of this work was possible thanks to the funding provided by the European Regional Development Fund and by the Consejería de Economía, Ciencia y Agenda Digital from Junta de Extremadura through grant GR18052.

## Conflict of Interest

The authors declare that the research was conducted in the absence of any commercial or financial relationships that could be construed as a potential conflict of interest.

## Publisher’s Note

All claims expressed in this article are solely those of the authors and do not necessarily represent those of their affiliated organizations, or those of the publisher, the editors and the reviewers. Any product that may be evaluated in this article, or claim that may be made by its manufacturer, is not guaranteed or endorsed by the publisher.
